# Biomaterial design for regenerating aged bone: materiobiological advances and paradigmatic shifts

**DOI:** 10.1093/nsr/nwae076

**Published:** 2024-02-28

**Authors:** Kai Dai, Zhen Geng, Wenchao Zhang, Xue Wei, Jing Wang, Guangjun Nie, Changsheng Liu

**Affiliations:** Engineering Research Center for Biomedical Materials of the Ministry of Education, East China University of Science and Technology, Shanghai 200237, China; State Key Laboratory of Bioreactor Engineering, East China University of Science and Technology, Shanghai 200237, China; Frontiers Science Center for Materiobiology and Dynamic Chemistry, East China University of Science and Technology; Shanghai 200237, China; Key Laboratory for Ultrafine Materials of the Ministry of Education, East China University of Science and Technology, Shanghai 200237, China; Institute of Translational Medicine, Shanghai University, Shanghai 200444, China; National Center for Translational Medicine (Shanghai) SHU Branch, Shanghai University, Shanghai 200444, China; Engineering Research Center for Biomedical Materials of the Ministry of Education, East China University of Science and Technology, Shanghai 200237, China; Frontiers Science Center for Materiobiology and Dynamic Chemistry, East China University of Science and Technology; Shanghai 200237, China; Engineering Research Center for Biomedical Materials of the Ministry of Education, East China University of Science and Technology, Shanghai 200237, China; Frontiers Science Center for Materiobiology and Dynamic Chemistry, East China University of Science and Technology; Shanghai 200237, China; Engineering Research Center for Biomedical Materials of the Ministry of Education, East China University of Science and Technology, Shanghai 200237, China; State Key Laboratory of Bioreactor Engineering, East China University of Science and Technology, Shanghai 200237, China; Frontiers Science Center for Materiobiology and Dynamic Chemistry, East China University of Science and Technology; Shanghai 200237, China; CAS Key Laboratory for Biomedical Effects of Nanomaterials & Nanosafety, CAS Centre for Excellence in Nanoscience, National Centre for Nanoscience and Technology, Beijing 100190, China; Center of Materials Science and Optoelectronics Engineering, University of Chinese Academy of Sciences, Beijing 100049, China; Engineering Research Center for Biomedical Materials of the Ministry of Education, East China University of Science and Technology, Shanghai 200237, China; Frontiers Science Center for Materiobiology and Dynamic Chemistry, East China University of Science and Technology; Shanghai 200237, China; Key Laboratory for Ultrafine Materials of the Ministry of Education, East China University of Science and Technology, Shanghai 200237, China

**Keywords:** materiobiology, biomaterial design, aging, bone regeneration, artificial intelligence

## Abstract

China's aging demographic poses a challenge for treating prevalent bone diseases impacting life quality. As bone regeneration capacity diminishes with age due to cellular dysfunction and inflammation, advanced biomaterials-based approaches offer hope for aged bone regeneration. This review synthesizes materiobiology principles, focusing on biomaterials that target specific biological functions to restore tissue integrity. It covers strategies for stem cell manipulation, regulation of the inflammatory microenvironment, blood vessel regeneration, intervention in bone anabolism and catabolism, and nerve regulation. The review also explores molecular and cellular mechanisms underlying aged bone regeneration and proposes a database-driven design process for future biomaterial development. These insights may also guide therapies for other age-related conditions, contributing to the pursuit of ‘healthy aging’.

## Teaser

This review exploresthe ongoing material design procedures involving screening of disordered biological functions, artificial intelligence-aided material `elements' selection, *in vitro* high-throughput fabrication and evaluation, and *in vivo* verification for clinical translation.

## INTRODUCTION

The seventh national population census indicates that China has transitioned into an aging society, with the population over 60 years old in Chinese mainland increasing to 18.70%, and the aging process of the population is far faster than that of many middle- and high-income countries [1]. The disease spectrum among the Chinese population, as well as in other countries around the world, has undergone a substantial shift. Specifically, the morbidity of aging-related diseases dramatically increases with the aging process [2]. Apart from stroke, ischemic heart disease, chronic obstructive pulmonary disease, various cancers, Alzheimer's disease, and hypertensive heart disease, diseases related to bone aging attract widespread attention due to their high prevalence and significant health impacts. In-depth investigation into the causes and treatment methods of bone aging-related diseases will reveal common patterns in aging, and facilitate the development of novel therapeutic approaches. This will also provide crucial insights for the diagnosis and treatment of other age-related diseases.

In aged individuals, bone regeneration capacity declines significantly, which increases the difficulty in treating aged bone injuries [3]. Multiple factors contribute to the decline in bone regeneration capacity of aged bone, including decreased self-renewal and differentiation capacity of mesenchymal stem cells (MSCs), excessive accumulation of inflammatory signals, impaired regeneration capacity of blood vessels, imbalanced bone anabolism and catabolism, and insufficient bone innervation [3–6]. Although biomaterials have improved the therapeutic effect for bone regeneration, this effect dramatically decreases when it comes to aged bone [7]. To address this dilemma, new biomaterial design strategies targeting aged bone regeneration should be proposed.

Materiobiology is a recently proposed scientific discipline for biomaterials design [8]. In aged bone repair, materiobiology focuses on the precise regulation and recovery of biological functions at the cellular, tissue, organ, and whole organism levels using functionalized biomaterials via the systematic combination of ‘elements’ from the biomaterial ‘toolbox’. The biomaterial ‘toolbox’ includes biochemical factors (e.g. growth factors, polypeptides, chemical and biological drugs, and genes) and customized biophysical effects (composition, mechanical properties, two-dimensional topography, three-dimensional geometry, as well as adequate delivery and fabrication technology).

**Figure 1.**
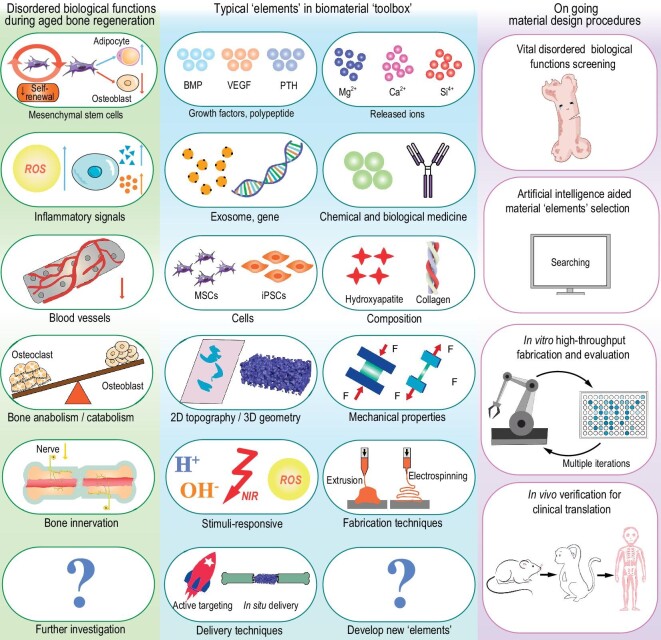
Illustration of biological function-targeted material design strategies. Beginning with the disordered biological functions inherent in the process of bone aging, and incorporating artificial intelligence to select suitable material ‘elements’, we aim to expedite the preliminary screening of biomaterials using an *in vitro* high-throughput fabrication and evaluation system. This process culminates in the validation of therapeutic efficacy through animal experiments and clinical trials. These material design procedures are anticipated to accelerate the pace of material development and clinical translation.

Figure 1 succinctly illustrates the current characteristics of aged bone regeneration, typical ‘elements’ in the biomaterial ‘toolbox’, and ongoing material design procedures. To better design biomaterials for ameliorating disordered biological functions in aged bone, the biomaterial ‘toolbox’ consisting of multiple types of essential ‘elements’ has been developed. Under the guidance of this material design strategy, the standard material design procedures could be concluded as follows: (1) choose vital disordered biological functions as therapeutic targets for bone repair in aged individuals; (2) given the characteristics of the selected biological functions, synergistically integrate ‘elements’ from the biomaterial ‘toolbox’ with the aid of artificial intelligence (AI); (3) optimize the ‘elements’ of modular biomaterials for aged bone regeneration through multiple iterations involving refining the composition and structure of the biomaterials using *in vitro* high-throughput fabrication and evaluation technologies; (4) conduct *in vivo* verification of the optimized biomaterials in small animals and non-human primates to advance clinical translation. Further optimization of this material design strategy mainly focuses on exploring new features of biological functions that hinder aged bone regeneration, as well as developing new ‘elements’ that target specific biological functions. Establishing a specialized database to pair specific biological functions with modular biomaterial ‘elements’ using artificial intelligence is a promising approach for expediting aged bone regeneration.

## BIOLOGICAL FUNCTION-TARGETED MATERIAL DESIGN STRATEGIES

### Stem cell manipulation-targeted material design strategies

In this section, we summarize recent material strategies targeting the regulation and activation of stem cells, such as mesenchymal stromal cells, whose migration, proliferation, and self-renewal capacities dramatically decrease in aged bone, hindering the bone regeneration process.

**Figure 2.**
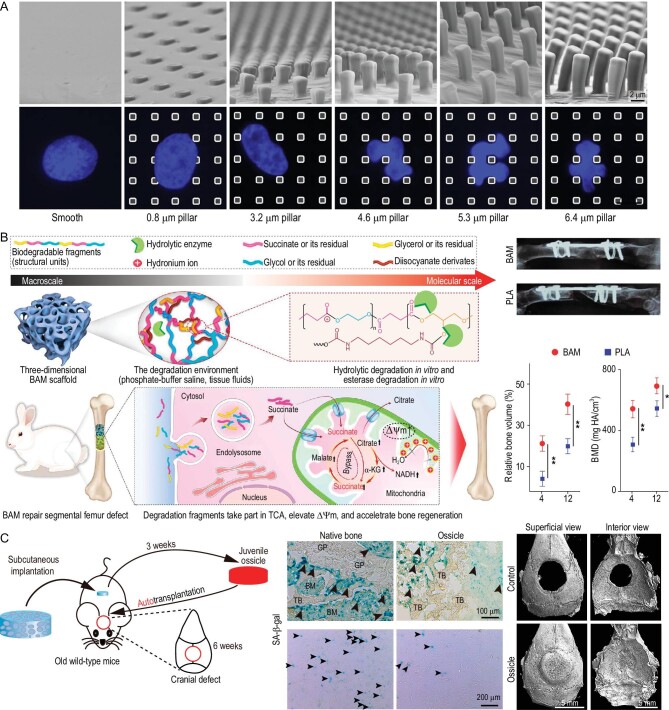
Material design strategies targeting the improvement of stem cell function. (A) Micropillars resulted in the self-deformation of cell nuclei and regulated stem cell differentiation. Reprinted with permission from [9]. Copyright © 2016 Elsevier BV. (B) Bioenergetic-active materials (BAM) scaffold elevated mitochondria metabolism level to accelerate bone formation via sustained release of energy-active units. The released energy-active units could be internalized and hydrolyzed to produce metabolic intermediates and finally enter the mitochondria to elevate mitochondrial membrane potential. Micro-computed tomography images and quantitative analyses (relative bone volume and bone mineral density (BMD)) confirmed that the BAM scaffolds accelerated bone generation compared to poly(lactic) acid scaffolds. TCA: tricarboxylic acid. Reprinted with permission from [10]. Copyright © 2020 American Association for the Advancement of Science. (C) Ossicles, induced by subcutaneous implantation of bone morphogenetic protein-2 (BMP-2)-loaded gelatin scaffolds in old mice for three weeks, exhibited juvenile phenotypes and enhanced cranial defect repair in old mice. Senescence-associated β-galactosidase (SA-β-gal) staining confirmed that the induced ossicles, as well as the stem cells derived from the ossicles, possessed fewer senescent cells compared to those in the native bone marrow. Micro-computed tomography images confirmed that the ossicles repaired the critical-sized cranial defect as soon as 6 weeks. Reprinted with permission from [11]. Copyright © 2020 Elsevier BV.

The physical properties of materials, such as their composition, degradability, 2D topography, and 3D geometry, can directly regulate stem cell behavior *in vitro* [12–14]. Ding *et al.* systematically studied how different surface micropatterns and matrix stiffness regulate stem cell fate using a well-designed micro-patterning technique [13]. They found that cell-cell contact significantly enhanced both osteogenic and adipogenic differentiation of rat bone marrow stem cells (BMSCs) [12]. Using the micro-patterning technique, they further identified that optimal adipogenic and osteogenic differentiation occurred in circular and star cells, respectively, which were linearly related to the cell perimeter [14]. Interestingly, they found that osteogenic differentiation was enhanced and adipogenic differentiation was alleviated on poly (lactic-co-glycolic acid) micropillared arrays with significant self-deformation of MSC nuclei. This revealed that nuclear deformation provided a new subcellular geometric cue to influence stem cell differentiation (Fig. 2A) [9].

Mooney *et al.* investigated the cell-matrix interaction in a synthetic hydrogel and confirmed that matrix rigidity had significant effects on clonally derived MSCs phenotype *in vitro* [15]. Specifically, 11–30 kPa was optimal for osteogenic differentiation and 2.5–5 kPa was optimal for adipogenic differentiation. They further demonstrated that hydrogels with tunable stress relaxation regulated stem cell fate *in vitro*, which were mediated by adhesion-ligand binding (such as Arg-Gly-Asp), actomyosin contractility, and mechanical clustering of adhesion ligands [16]. Inspired by these findings, in recent years our group has developed a series of poly (glycerol sebacate) (PGS)-based elastic materials with excellent biocompatibility and adjustable biodegradability for tissue regeneration [17,18]. We utilized the click chemistry method to synthesize injectable and viscoelasticity-adjustable polyhydroxy PEGylated poly (glycerol sebacate) (PEGS-OH) hydrogels for regenerating rabbit articular chondral defects and rat calvarial defects, respectively [17]. Previous studies conducted by our group have confirmed that mesoporous bioactive glass (MBG)-, β-tertiary calcium phosphate (β-TCP)-, and calcium phosphate cement-based biomaterials exhibited excellent osteoinductive capacity, thereby promoting critical bone repair in rabbit femur cavity defects and rat calvarial defects models [19,20]. However, the inherent brittleness and generally low mechanical strength of these biomaterials hampered their clinical application. Our group further improved the mechanical strength of these biomaterials via the organic/inorganic composite strategy. In a recent paper published by our group, we combined viscoelastic low-crosslinked PEGS with MBG to construct a difunctional PEGS/MBG bilayer scaffold for rabbit full-thickness osteochondral defect repair [18]. The viscoelastic PEGS layer significantly stimulated chondrogenic differentiation, maintained chondrocyte phenotype, and enhanced cartilage matrix secretion, while the MBG layer promoted the osteogenesis of recruited MSCs.

The metabolic dysfunctions of stem cells within aged bones hinder the process of bone regeneration [21]. Developing innovative biomaterials that regulate and reset disordered cellular bioenergetics could be a promising approach to promoting aged bone regeneration. Zhang *et al.* reported on BAM that enhanced bone regeneration by modulating cellular metabolic states (Fig. 2B) [10]. This bioenergetic-active material was composed of energy-active units, and the degradation fragments were internalized by cells and entered the mitochondria to participate in the tricarboxylic acid cycle, which enhanced the cellular metabolic state and resulted in robust bone regeneration in a rabbit hindlimb segmental defect model. Previous studies have confirmed that the concentration of citrate in bone and plasma is reduced in osteoporosis, and citrate, as an osteopromotive factor, supports the osteogenic differentiation of MSCs via regulation of energy-producing metabolic pathways [22]. The Yang group previously synthesized citrate-based tannin-bridged bone composites by modifying hydroxyapatite with tannic acid and covalently bridging immobilized tannic acid with the citrate-based biodegradable polymer [23]. The rabbit lumbar fusion model confirmed that the porous citrate-based tannin-bridged bone composites possessed excellent osteoconductivity and osteoinductivity.

Recently identified mouse and human skeletal stem cells are essential for bone development and regeneration [24,25]. In aged mice, senescent skeletal stem cells generate an inflammatory microenvironment that reduces callus volume and impairs bone fracture healing [26]. BMP-2 is a widely used growth factor in clinical bone defect repair [27]. The Longaker group reactivated aged mice skeletal stem cells, ameliorated the inflammatory microenvironment, and finally enhanced bone fracture repair using a BMP-2/colony-stimulating factor-1 antibody-loaded photo-crosslinked polyethylene glycol (PEG)/mercaptoacetic ester hydrogel [26]. Since the BMP-2-loaded scaffold can trigger endochondral ossification ectopically, our group repaired critical cranial defects in aged mice via the transplantation of juvenile ossicles induced by BMP-2-loaded gelatin sponges (Fig. 2C) [11]. This *in vivo* pre-incubation strategy creates a juvenile environment and reactivates the residual regenerative capacity of individuals. At the same time, the materials involved in this strategy have been widely used in clinical practice, and it is very likely to achieve clinical translation after the necessary large animal validation.

Beyond the physical properties of the biomaterial, directly modulating the energy metabolism process of stem cells by utilizing the degradation products of the biomaterial holds promise as a strategy for rejuvenating aged bones. Additionally, the *in vivo* pre-incubation strategy, employing bioactive factors to effectively stimulate the body's regenerative capacity, represents a therapeutic approach with significant potential for clinical translation.

### Inflammatory microenvironment regulation-targeted material design strategies

The dysregulation of inflammatory cells and increased levels of inflammatory signals, such as overactive proinflammatory macrophages and consistently high levels of reactive oxygen species (ROS), impair the regenerative capacities of aged bone [3,30]. In this section, we summarize recent material strategies that target the regulation of the inflammatory environment.

**Figure 3.**
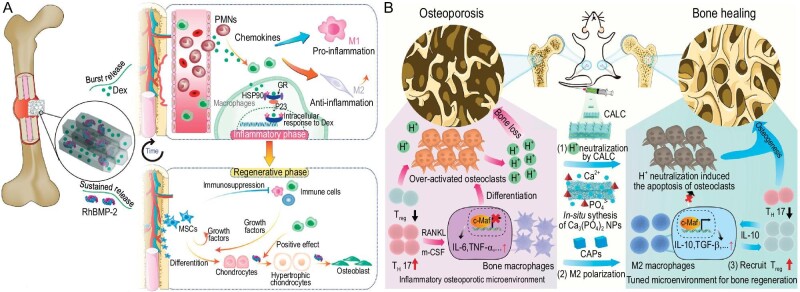
Material design strategies targeting the regulation of inflammatory microenvironment. (A) Porous mesoporous bioglass scaffolds achieved sequential release of dexamethasone and BMP-2. The burst release of dexamethasone at the initial stage inhibited the overactive inflammatory response and subsequently promoted the sustained-release BMP-2-induced endochondral ossification. Reprinted with permission from [28]. Copyright © 2021 Wiley-VCH Verlag GmbH & Co. (B) Calcium-aluminum-layered double hydroxide (CALC) nanosheets mediated the apoptosis of osteoclasts via neutralizing excessive protons and degraded to release calcium ions for the generation of calcium phosphate nanoparticles. Newly generated calcium phosphate nanoparticles switched from M1 macrophages to M2 macrophages in osteoporotic bone and subsequently recruited regulatory T cells and suppressed T helper 17 cells. Finally, such an inorganic nanomaterial-based strategy altered the bone immune microenvironment and significantly increased the bone volume in an aged mouse model. Reprinted with permission from [29]. Copyright © 2022 American Chemical Society.

Innate or adaptive immune cells, such as macrophages and lymphocytes, play a crucial role in bone aging and regeneration [31,32]. Diverse biomaterials can further regulate the phenotype and number of inflammatory cells. Our group confirmed that scaffolds with hierarchical macro-micro-mesoporous scaffolds promoted M1 polarization at an early stage (0–3 days) and M2 polarization at a later stage (3–7 days) after implantation in the thigh muscle pouches of mice [33]. Niu *et al.* developed a mono-compositional and anisotropic porous scaffold to mediate full-range and endogenous bone regeneration by accurately driving macrophages to a pro-regenerative phenotype in a rat calvarial defect model [34]. In a recent study, our group discovered that dexamethasone, a classic anti-inflammatory agent, modulated the osteogenic potential of BMP-2 in a dose-dependent manner [28]. Within a porous mesoporous bioglass scaffold, the burst release of dexamethasone created an immunosuppressive environment and enhanced the osteochondral differentiation capacity of MSCs (Fig. 3A).

The role of adaptive immune cells, such as T cell, in bone regeneration is receiving increased attention form researchers [32]. Ono *et al.* reviewed recent biological progress regarding the impact of adaptive immune cells on bone regeneration. They highlighted the promotive effect on bone healing of interleukin (IL)-17-producing γδ T cells, as well as the inhibitory effect on bone regeneration of CD8^+^ T cells [35]. Furthermore, the impact of IL-10-producing B cells on bone healing may involve the suppression of excessive and/or prolonged inflammation. Building on these recent discoveries, Yu *et al.* developed biomimetic hydroxyapatite nanorods that promoted the osteogenesis of MSCs through T cell-derived IL-22 in a mouse mandibular defect model [36]. Hotchkiss *et al.* confirmed that increasing the roughness and surface wettability of titanium implants promoted the development of a pro-regenerative T-helper 2 response after implantation in the femur of male mice [37].

Excessive accumulation of inflammatory signals in aged bone impairs bone healing, and biomaterials targeting inflammatory signal-scavenging provide a promising solution for this issue [38]. An acid environment (pH ∼4), resulting from overactive bone resorption triggered by mature osteoclasts, is a typical characteristic of osteoporosis [39]. Fu *et al.* achieved effective osteoporosis reversion using calcium-aluminum layered double hydroxide through acid neutralization and immune regulation in aged mice (Fig. 3B) [29]. Elevated levels of ROS are another characteristic of aged bone [7]. Zhou *et al.* fabricated a mussel-inspired electroactive, and osteoinductive porous titanium scaffold that could persistently scavenge ROS and enhance rabbit femur defect repair [40].

The current research on material design for modulating the inflammatory microenvironment is centered on the phenotypic transformation of pro-inflammatory macrophages and the efficient clearance of high-level ROS. It is imperative to acknowledge that the dysregulation of adaptive immunity significantly impedes the repair mechanisms of aged bone tissue, which should be a critical consideration in future material design.

### Blood vessel regeneration-targeted material design strategies

Insufficient blood supply and revascularization capacity in aged bones hinder efficient bone regeneration [45]. The application of biomaterials, incorporated with pro-angiogenic factors or biomimetic structure, benefit the regeneration of aged bone. In this section, we summarize recent material strategies that target blood vessel regeneration.

**Figure 4.**
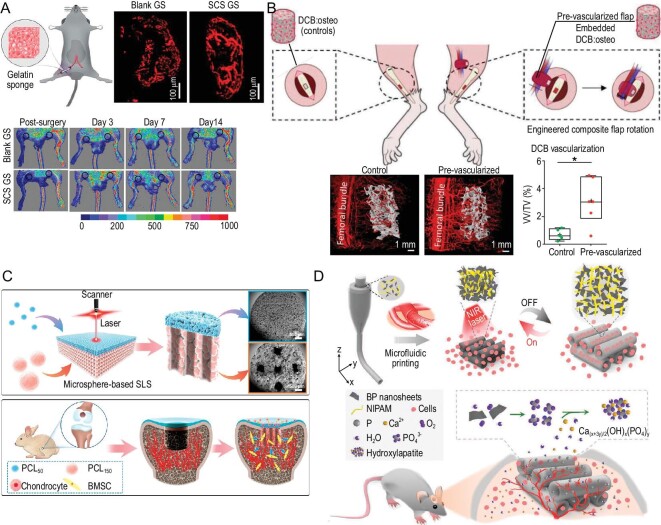
Material design strategies targeting the regulation of blood vessels. (A) Semisynthetic sulfated chitosan (SCS) induced angiogenesis in mouse ischemia. Reprinted with permission from [41]. Copyright © 2020 American Association for the Advancement of Science. (B) *In vivo* engineered composite flaps underwent a pre-vascularization stage *in vitro* and were subsequently incubated *in vivo* for maturation. The composite flaps were finally transplanted to the bone defect area and supported mouse long-term bone repair. DCB: decellularized bone matrix. Reprinted with permission from [42]. Copyright © 2021 Wiley-VCH Verlag GmbH & Co. (C) poly(ε-caprolactone) (PCL) microsphere-based scaffolds with a dense cartilage zone and porous subchondral bone zone were fabricated via a selective laser sintering technique. The integrated scaffolds enhanced angiogenesis of the subchondral bone zone and facilitated osteochondral defect repair in rabbits. Reprinted with permission from [43]. Copyright © 2022 Elsevier BV. (D) Microfluidic 3D printing responsive scaffolds incorporated with black phosphorus (BP) exhibited a reversible shrinkage and swelling behavior that could form photothermal responsive channels. The embedded BP nanosheets could be further oxidized to provide phosphorus oxides for *in situ* biomineralization. A mouse cranial defect model confirmed that the fabricated responsive scaffolds accelerated vascularization and osteogenesis in the bone defect area. Reprinted with permission from [44]. Copyright © 2021 Wiley-VCH Verlag GmbH & Co.

Classic pro-angiogenic factors, such as vascular endothelial growth factor (VEGF) and fibroblast growth factor, have been widely utilized in bone repair and regeneration. However, their overuse can result in abnormal angiogenesis [46,47]. Our group recently found that semisynthetic SCS readily engaged anti-inflammatory macrophages and increased their secretion of endogenous VEGF to induce angiogenesis in mice hindlimb ischemia (Fig. 4A) [41]. Our group further designed a SCS-functionalized dual-modular growth factor delivery scaffold composed of BMP-2- and SCS-loaded MBG matrix with hollowed channels filled with VEGF- and SCS-loaded gelatin methacryloyl (GelMA) hydrogel [48]. This dual-modular scaffold exhibited stable vascularity and a high degree of bone formation.

Osteocutaneous flap transplantation has been used clinically for repairing composite tissue defects [49]. However, due to the limited donor sites available clinically, the *in vivo* osteocutaneous flap prefabrication strategy could serve as a potential alternative solution [50]. Redenski *et al.* fabricated a composite flap with its own vascular supply *in vivo* using synthetic soft-tissue matrices and decellularized bone (Fig. 4B) [42]. The addition of dental pulp stem cells and human adipose microvascular endothelial cells further enhanced the vascular development of the implant. *In vivo* mice models confirmed that the fabricated composite flap repaired complex tibia defects and bridged muscle defects. Our group found that subcutaneously implanted BMP-2-loaded gelatin scaffolds underwent endochondral ossification and identified a periosteum-like tissue at the early stage of endochondral ossification in an aged mice model [51]. This periosteum-like tissue was highly vascularized and abundant in platelet-derived growth factor receptor-α^+^ periosteum-derived stem cells. The critical cranial defect model of aged mice confirmed that the induced autologous periosteum-like tissue efficiently repaired the defect zone. Baldwin *et al.* developed an *in vitro* pre-vascularized construct using PCL tubular scaffolds incorporated with human endothelial and bone marrow mesenchymal stem cells [52]. This *in vitro* pre-vascularized construct was structurally similar to periosteum and efficiently repaired NOD-SCID gamma mice femur defects.

Biomimetic channel structures are beneficial for initial vascularization and subsequent osteogenesis [53]. Gu *et al.* fabricated PCL microsphere-based osteochondral scaffolds for rabbit cartilage and subchondral bone regeneration (Fig. 4C) [43]. These osteochondral scaffolds were composed of a dense phased-cartilage zone for the inhibition of vessel invasion, as well as a porous phased-subchondral bone zone for the ingrowth of bone and vessels. Moreover, channels were fabricated by selective laser sintering technology. Wang *et al.* utilized microfluidic 3D printing technology to fabricate black phosphorus-doped fibrous scaffolds with photothermal responsive channels for vascularized bone regeneration in mice cranial defect models (Fig. 4D) [44]. These porous scaffolds displayed reversible deformation capacity triggered by near-infrared (NIR) irradiation, as well as accelerated *in situ* biomineralization.

The efficient regeneration and reconstruction of vasculature is essential for the repair of aged bones. The strategic incorporation of classical pro-angiogenic factors into materials has proven effective at enhancing vascular regeneration. In addition to the existing classical angiogenic factors, identifying additional molecules with angiogenic activity could further enrich the types of elements within the biomaterial ‘toolbox’. Notably, the chemical modification of naturally derived macromolecules represents a significant avenue worthy of attention. Furthermore, the focal point for future research lies in the development of customized controlled-release systems for releasing angiogenic molecules or cells. These systems should meet the regeneration needs of specific vasculature for various types of aged bone repair, such as fractures and osteochondral defects.

### Bone anabolism and catabolism intervention-targeted scaffold design strategy

Active bone catabolism accompanied by declining bone anabolism results in various bone diseases such as osteoporosis, bone nonunion, or delayed union in aged bones. Multiple types of drugs or biomaterial systems have been developed to address these issues. In this section, we summarize recent material strategies that target bone anabolism and catabolism intervention.

Calcium phosphate-based materials are widely used for bone repair, with previous research mainly focusing on their bone-promoting abilities rather than their role in bone resorption regulation. Our group recently discovered that the calcium-to-phosphate ratio in these materials played a pivotal role in regulating osteoclast-mediated osseointegration [58]. Specifically, an excessive release of phosphate ions released from calcium phosphate-based materials with a low calcium-to-phosphate ratio had a negative effect on osteoclast formation by inhibiting the binding of receptor activator for nuclear factor-κ B ligand to its receptor during the repair of rat cranial defects.

Various bisphosphonates have been clinically approved for suppressing the overactive behavior of osteoclasts. Quan *et al.* fabricated a coral-like hydroxyapatite that was synthesized by spontaneous assembly of alendronate and Fe_3_O_4_ onto hydroxyapatite nanocrystals without inducing lattice deformation, for bone regeneration in osteoporotic rats [59]. This coral-like hydroxyapatite displayed a proper magnetic property as well as a controlled release pattern. Moreover, the overactive behavior of osteoclasts creates a low pH environment in the osteoclast microenvironment, a phenomenon also present in osteoarthritis. In response to this specific pathological microenvironment, our group designed a bisphosphonate-conjugated nano-apatite system with pH-responsive capacity [60]. This nano-apatite system efficiently inhibited abnormal subchondral bone remodeling and delayed osteoarthritis progression in rats.

**Figure 5.**
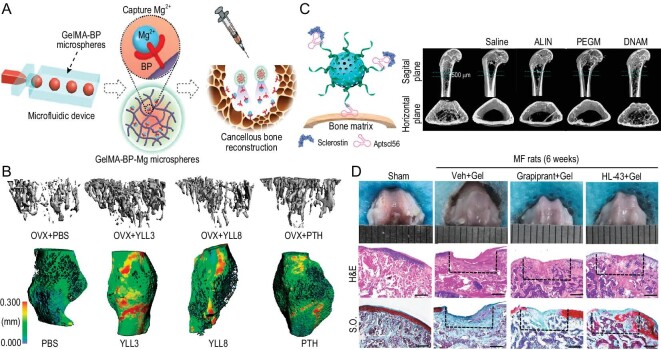
Material design strategies targeting the intervention of bone anabolism and catabolism. (A) Bisphosphonate-functionalized injectable hydrogel microspheres were fabricated using coordination reaction and microfluidic techniques. The long-term released Mg^2+^ promoted osteogenesis, while the released bisphosphonate inhibited osteoclast activities. The *in vivo* osteoporotic rat model confirmed that the injectable microspheres could efficiently enhance cancellous bone regeneration. Reprinted with permission from [54]. Copyright © 2021 American Chemical Society. (B) A combinational peptide screening strategy was developed for rapid discovery of osteogenic progenitor cell-targeted peptides. Two lead compounds, YLL3 and YLL8, were confirmed to improve trabecular bone formation and accelerate bone healing in mice. Reprinted with permission from [55]. Copyright © 2020 Springer Nature Limited. (C) A bone targeting nanomedicine containing PEGylated dendritic mesoporous silica nanoparticle and anti-sclerostin DNA aptamer Aptscl56 layer was fabricated for osteoporosis treatment. This bone-targeted drug delivery was confirmed to improve trabecular bone formation and accelerate bone healing in ovariectomized mice. Reprinted with permission from [56]. Copyright © 2022 Elsevier BV. (D) An injectable starch hydrogel containing HL-43, a novel selective prostaglandin E receptor 4 antagonist, was developed for repairing rat osteochondral defect. HL-43 enhanced cartilage anabolism and accelerated articular cartilage formation. Reprinted with permission from [57]. Copyright © 2022 Springer Nature Limited.

In the quest to enhance bone regeneration, particularly in aging bones, the suppression of overactive osteoclasts is crucial, but it is equally vital to augment osteogenesis. Research has highlighted the role of metal cations in promoting osteogenic effects. Zhao *et al.* employed a microfluidic device to create an injectable hydrogel microsphere that was modified with bisphosphonate and incorporated Mg^2+^ (Fig. 5A) [54]. This novel microsphere facilitated the repair of bone defects in osteoporotic rats, demonstrating the potential of metal cations in bone regenerative medicine.

The osteoinductive protein BMP-2, commonly used in clinical settings, has been found to have an enhanced effect on bone formation when used in conjunction with bisphosphonates. This synergistic effect has been substantiated through various studies. For instance, Bosemark *et al.* showed that systemic bisphosphonate treatment significantly improved the mechanical properties and bone density of BMP-2 induced calluses in a rat fracture model [61]. Further advancing the field, Kootala *et al.* developed bisphosphonate-functionalized hydrogels that could regulate BMP-2 binding and its subsequent release. This innovation proved instrumental in the rapid differentiation of progenitor cells *in vitro*, showcasing a promising avenue for enhancing bone regeneration strategies [62]. Moreover, Houdt *et al.* addressed a critical-sized segmental femoral bone defect in a rat model using a combination of self-healing calcium phosphate nanoparticles, bisphosphonate-conjugated hyaluronan, and a supplementary administration of low-dose BMP-2. This multifaceted approach resulted in successful treatment outcomes [63].

Peptides that promote bone anabolism have also been introduced into the design of materials to enhance the regeneration of aging bone. Our group developed a NIR light-responsive material system to release bone anabolism proteins, such as parathyroid hormone (PTH), at a low and effective concentration [64]. NIR laser irradiation triggered the gel-sol transition of photothermal-responsive microspheres, which not only achieved on-demand PTH release but also facilitated the formation of micropores *in situ* [64]. Complementing this approach, Jiang *et al.* developed a combinational peptide screening strategy to rapidly identify osteogenic progenitor cell-targeted peptides (Fig. 5B) [55]. With this strategy, they discovered two lead compounds, YLL3 and YLL8. Both compounds demonstrated strong pro-osteogenic capacities *in vitro* and were able to augment bone formation in mice, indicating their potential as therapeutic agents for bone regeneration.

Romosozumab, a pioneering humanized antibody that antagonizes sclerostin, simultaneously augments bone formation and attenuates bone resorption. It exhibits a pronounced enhancement of bone density and mass in the lumbar spine and hip regions of postmenopausal women when compared to PTH treatments [65]. Niu *et al.* have ingeniously developed aptamer-functionalized nanoparticles that target bone tissue, sequestering sclerostin *in situ*, which has resulted in a substantial elevation of bone mass in ovariectomized mice (Fig. 5C) [56].

Concomitant research has unveiled alternative methodologies for modulating osteoclast activity. Various phytomolecules, including icaritin and asperosaponin VI, sourced from traditional Chinese medicinal practices, exhibit potent inhibition of osteoclastogenesis, thereby offering a viable defense against osteoporosis [66,67]. Huang *et al.* designed a bone-targeting oligopeptide-modified liposome to precisely deliver icaritin for preventing osteoporosis in mice [68]. Tu *et al.* have confirmed that the suppression of cyclooxygenase-2 expression within subchondral bone could ameliorate the clinical manifestations associated with osteoarthritis or rheumatoid arthritis [69]. In a novel discovery, the Luo group has discerned that prostaglandin E2, a major cyclooxygenase-2 product, induces the activation of prostaglandin E receptor 4 in osteoclasts located within subchondral bone. This process is implicated in the regulation of osteoarthritis. They have further identified a small molecule antagonist of this receptor, designated HL-43 [57]. Employing starch hydrogels imbued with HL-43, they have successfully facilitated cartilage regeneration in a rat model presenting with cartilage defects induced by microfracture surgery (Fig. 5D) [57].

In the design of materials aimed at promoting bone regeneration in aging, it is crucial to balance the inhibition of excessive osteoclastic activity with the enhancement of osteogenic capacity. The incorporation of appropriate bioactive substances that influence bone anabolism and catabolism, such as peptides and neutralizing antibodies, is a preferred strategy in material design. Furthermore, the utilization of advanced delivery systems for targeted bone delivery will be an important direction for future research.

### Nerve regulation-targeted scaffold design strategy

Innervation within the bone is crucial for the maintenance of a balanced differentiation function between osteogenesis and adipogenesis in bone marrow stromal cells (BMSCs), as well as for the energy metabolism equilibrium between bone and adipose tissue [74,75]. The aging process in bones leads to a significant reduction in the expression of the prostaglandin E receptor 4 sensory nerve receptor, resulting in an impaired ability to effectively perceive changes in bone metabolism [76]. In this section, we summarize recent material strategies that target nerve regulation.

**Figure 6.**
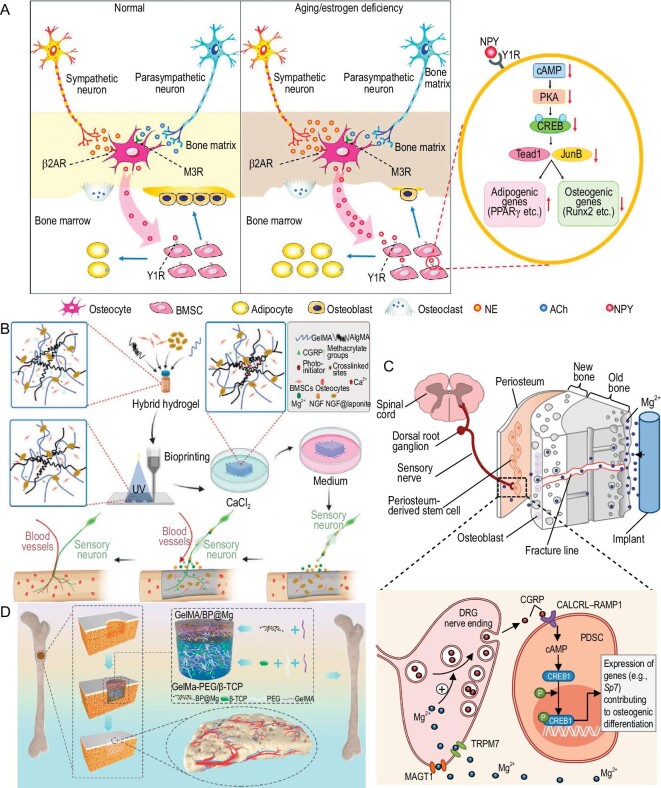
Material design strategies targeting nerve regulation. (A) The schematic diagram illustrates that the autonomic nervous system increases the production of neuropeptide Y (NPY) in osteocytes, and overexpression of NPY subsequently results in an imbalance between adipogenesis and osteogenesis of BMSCs in aged or estrogen-deficient mice. Reprinted with permission from [70]. Copyright © 2021 Wiley-VCH Verlag GmbH & Co. (B) A 3D-printed scaffold containing nerve growth factor (NGF) was fabricated to promote innervation during bone repair in rats. The sustained release of NGF from the scaffold stimulated sensory neurons to secrete calcitonin gene-related polypeptide (CGRP). The elevated expression of CGRP ultimately enhanced the osteogenic differentiation of BMSCs and accelerated bone regeneration. Reprinted with permission from [71]. Copyright © 2022 Wiley-VCH Verlag GmbH & Co. (C) Magnesium ions derived from implants diffuse to the periosteum area and stimulate the dorsal root ganglia (DRG) to sequentially release CGRP. The released CGRP further increases the expression of osteogenic genes and ultimately promotes bone fracture healing in osteoporotic rats. Reprinted with permission from [72]. Copyright © 2016 Springer Nature Limited. (D) A bilayer hydrogel composed of an upper layer containing magnesium-ion-modified black phosphorus nanosheets and a bottom layer containing β-TCP promoted the formation of nerve-vascular networks at the periosteum area and ultimately enhanced osteogenic differentiation in a rat calvarial defect model. Reprinted with permission from [73]. Copyright © 2022 Elsevier BV.

The pivotal role of sensory nerve innervation in the homeostasis of bone during bone repair has been widely underestimated. Recent studies have verified that various neuropeptides, including NPY, CGRP, neurotrophin-3, and substance P, are implicated in the modulation of bone regeneration and aging [6,70]. Zhang *et al.* elucidated that the autonomic nervous system governs the secretion of osteocyte-derived NPY, and that an overproduction of NPY can disrupt the equilibrium between adipogenic and osteogenic differentiation in BMSCs in both aged and estrogen-deficient mice. (Fig. 6A) [70].

CGRP has been identified as a regulator of macrophage polarization, local blood flow, and osseointegration at sites of bone defects [77]. Yu *et al.* demonstrated that BMSCs genetically modified to produce CGRP exhibited an enhanced osteogenic differentiation capacity *in vitro* [78]. In addition, neurotrophin-3 has been observed to increase BMP-2 and VEGF expression at damaged growth plates, thereby fostering osteochondral repair in rats [79]. Moreover, Liu *et al.* reported that the osteogenic differentiation of human dental MSCs could be regulated by NGF via the key epigenetic regulator KDM4B [80]. Li *et al.* engineered a 3D-printed scaffold designed to replicate the ossification center environment, which supports innervation in bone regeneration (Fig. 6B) [71]. This biomimetic scaffold facilitates the sustained release of NGF, promoting the formation of a neurovascular network and the osteogenic differentiation of BMSCs during the repair of cranial defects in rats. The role of substance P in bone regeneration has been documented in the literature. Amirthalingam *et al.* created a chitin/poly (lactic-co-glycolic acid)-CaSO_4_-based hydrogel, verifying that incorporating lactoferrin and substance P enhanced bone regeneration during the repair of critical-sized cranial defects in mice [81]. Introducing neuropeptides in material design has been proven to be an effective strategy for enhancing neuroregulation in bone repair.

Biodegradable magnesium-based orthopedic implants have been efficaciously deployed for fracture repair, yielding favorable therapeutic outcomes. [82]. Zhang *et al.* revealed the essential effect of a substantial increase in CGRP induced by magnesium implants in augmenting osteogenic differentiation using a rat bone-fracture model (Fig. 6C) [72]. The dissolution of Mg^2+^ from these implants led to an elevated release of CGRP from the dorsal root ganglia (DRG), which, upon engaging with CGRP receptors on periosteal stem cells, initiated the phosphorylation of cAMP-responsive element-binding protein 1 (CREB1) through the cAMP pathway, thereby enhancing the transcription of osteogenic genes. This investigation proposes that the osteogenic influence exerted by magnesium-based implants is contingent upon sensory innervation. Beyond magnesium-based implants, Yang *et al.* substantiated that whitlockite, a natural magnesium-containing bone mineral, could concurrently stimulate osteogenic and neural activities both *in vitro* and *in vivo* using a rat cranial defect model [83].

Multiple studies have validated the efficacy of a periosteum-mimetic strategy in achieving neural integration within the bone repair process. Xu *et al.* devised a bilayer hydrogel with an upper periosteal-repair layer composed of magnesium-ion-modified BP nanosheets and GelMA, and a lower layer composed of PEG-modified GelMA and β-TCP (Fig. 6D) [73]. This stratified-structural hydrogel promoted the formation of a periosteal nerve-vascular network, significantly bolstering bone regeneration in a rat model with a critical-sized cranial defect. Similarly, Wan *et al.* employed a periosteum-mimetic strategy, fabricating an eggshell-like membrane incorporated with cerium oxide (Ce-ESM) to boost bone regeneration as well as neuro-vascularization during the repair of cranial defects in mice [84]. The cerium released from Ce-ESM induced a phenotypic shift in macrophage lineage cells towards tartrate-resistant acid phosphatase-positive pre-osteoclasts, which further supported neuro-vascularization and bone formation through the modulation of slit guidance ligand 3, VEGF, and platelet-derived growth factor-BB.

The Cao group conducted a systematic investigation into the regulatory role of the nervous system in bone regeneration, introducing the concept of ‘skeletal interoception’ and highlighting the significance of neural regulation in bone aging [76]. As research delves deeper into areas such as neural modulation of bone homeostasis and differentiation of bone marrow stromal cells, additional targets aimed at aged bone regeneration are anticipated to be discovered. These insights offer valuable perspectives for subsequent material design, enriching the elements within the biomaterials ‘toolbox’. Particularly in developing materials capable of intervening in systemic bone aging, significant breakthroughs are expected in the future.

## SUMMARY AND OUTLOOK

In this review, we concentrate on biological function-targeted material design strategies to enhance aged bone regeneration, encompassing stem cell manipulation, inflammatory microenvironment regulation, blood vessel regeneration, bone anabolism and catabolism intervention, and nerve regulation. We underscore the precise modulation of crucial and central biological functions during aged bone regeneration using a combination of ‘elements’ from the biomaterial ‘toolbox’. Specifically, we propose a concept that emphasizes directly augmenting the biological functions necessary for aged bone regeneration through the modular design and assembly of functional ‘elements’ of bioactive materials, such as the synergistic integration of three-dimensional geometry, growth factors, and appropriate delivery technology. In addition to the current advancements in biological function-guided material design strategies, we must further expand the variety of ‘elements’ in the biomaterials ‘toolbox’ to more effectively address the challenges in aged bone regeneration.

A high proportion of senescent cells in aged individuals is strongly associated with numerous age-related diseases [85]. Strategies to eliminate senescent cells have been employed to alleviate age-related symptoms [86]. Senolytic chimeric antigen receptor (CAR) T cells have been developed to reverse senescence-associated pathologies [87]. Prior research has shown that aged skeletal stem cells create an inflammatory degenerative niche that impedes fracture repair [88]. The aged senescent cells in the callus also hinder fracture repair. The removal of bone-related senescent cells could be a promising avenue for enhancing aged bone regeneration. Significant advancements in precision medicine offer a range of novel therapeutic methods, including CAR T cells and antibody-drug conjugates, which may considerably enrich the types of ‘elements’ in the biomaterial ‘toolbox’. To precisely eliminate bone-related senescent cells, we should develop an advanced bone-targeting system and identify specific senescent cell features, such as senescence-specific surface antigens and high levels of SA-β-gal [89]. By synergistically combining these ‘elements’, we could create a series of intelligent biomaterials to efficiently enhance aged bone regeneration and even alleviate the senescent pathologies of the entire body's skeletal system.

A thorough investigation of the molecular and cellular networks regulating aged bone regeneration could provide new insights for the development of novel ‘elements’. Emerging evidence confirms that bone participates in the energy metabolism of various tissues, such as adipose tissue, liver, pancreas, testis, and brain, through bone-derived factors [21]. Simultaneously, several studies have identified that the brain [90], nervous system [70,77], epididymal adipose [91], and gut bacteria [92] play roles in bone regeneration and development. By further dissecting the specific signaling pathways and cell subsets involved in the complex crosstalk between bone and multiple tissues using multi-omics approaches, researchers may identify new biological functions that could serve as targets for biomaterial design.

High-throughput *in vitro* platforms, such as organoids, which can assess the regulatory effects of biomaterial ‘elements’ on specific biological functions, will significantly expedite research progress [93]. Bone is a sophisticated organic-inorganic composite material, while the bone niche is a complex system involving cells derived from different germ layers [94,95]. Currently, several groups have developed a series of bone organoids for investigating bone development and drug screening. Most of these bone organoids consist of only two or three cell types (e.g. MSCs, osteoclasts, or endothelial cells) and hydrogel scaffolds (e.g. gelatin hydrogel), which cannot accurately mimic native bone and bone marrow niches [96]. Constructing more biomimetic bone organoids to realistically evaluate biomaterial ‘elements’ and accelerate multiple iteration processes are the most pressing challenges.

The construction of a high-throughput fabrication and evaluation system for biomaterials, utilizing artificial intelligence technology, is a pivotal component within the overall design strategies. Presently, deep-learning models have been demonstrated to significantly enhance the efficiency of discovering stable inorganic crystals [97]. Artificial intelligence-driven platforms facilitate the effective integration of computations, historical knowledge, and robotics [98]. They are also capable of proactively extracting improvement clues from unsuccessful syntheses, thereby advancing the technology of materials screening and synthesis design. At the same time, transformer-based large language models have also been employed for the automated synthesis of complex compounds [99]. These advancements bolster our confidence in the high-throughput fabrication of biomaterials powered by artificial intelligence. Moreover, the substantial progress in image processing through artificial intelligence has gradually made the high-throughput evaluation of the biological functions of biomaterials feasible [100].

The development of an aged bone repair-associated database will effectively enhance the matching between designed biomaterials and their therapeutic effects on diseases, significantly accelerating the speed of clinical translation. This specialized database will be constructed based on the scientific categorization of metadata extracted from an extensive compendium of primary documents that link specific biological functions to their corresponding biomaterial ‘elements’. In the future, an ideal biomaterial design process for aged bone regeneration will involve identifying scientific inquiries, retrieving specific biological functions, locating matched biomaterial ‘elements’ within a biomaterials database, selecting candidate material combinations through a high-throughput fabricating and screening system, validating the efficacy of biomaterials via *in vivo* experiments, and ultimately achieving clinical application following clinical trials.

Materiobiology, as an interdisciplinary field, directs biological function-targeted material design strategies that require collaboration from multidisciplinary researchers to further enrich its research content and methods. We seek to drive a paradigm shift in the research approach to material design for bone aging-related diseases and to inspire a similar shift in the research paradigm for other aging-related diseases. This will facilitate the faster and more effective development of interventions for aging-related diseases, benefiting a larger patient population and bringing the vision of ‘healthy aging’ closer to reality for more individuals.
